# This Week in Parliament

**Published:** 1970-10

**Authors:** John Clutton-Brook


### This Week in Parliament

**From Our Parliamentary Correspondent, Thursday, 27th June 2070**

As this was the eighteenth anniversary of the dissolution of the House of Commons, the proceedings started with a short introductory speech from Lord Evans, now Chairman of the Life Peers. Readers of this column will no doubt be aware that Lord Evans, as Secretary, and now only member of the Amalgamated Trade Unions, has recently ascended to the Throne on the death of Lord Williams, our beloved Monarch for so many years. His Lordship described the continuing success of the present form of government, which has so effectively resolved all the vexed problems of party politics. He finished his distinguished oration by welcoming Lord Higgins, recently appointed to the peerage for his outstanding work on criminal reform.

The House then discussed a suggestion put forward by Lord Henderson, Shop Steward for the South-west Region, that the weekly working day should be changed from Thursday to Wednesday. Lord Henderson said that he believed that the majority of his constituents would prefer Wednesday, as they would then come back to work one day earlier after their weekend's recreation. Lord Higginbotham, Shop Steward for Yorkshire, remarked that he thought that the suggestion showed distinct reactionary tendencies and some muddled thinking. The Chairman said that his remark about muddled thinking did not accord with the dignity of the House and that he should withdraw it. Lord Higginbotham, with an obscure reference to a "duftayporth", believed to be an old British coin worth about £0.002, agreed to do so. The House finally decided that there was insufficient evidence to warrant such a change.

Lord Sneath, Shop Steward for the Greater London Area, said that he was having considerable trouble with a large group of Bohemian artists in Chelsea, who were consistently working on their pictures for more than one day a week. The explanation give for this was that what they were doing was recreation rather than work. Lord Sneath pointed out that, since the pictures were obviously designed to be hung on a wall, they were in competition with the plasterers and decorators. He said, further, that the usual penalty for working more than one day a week, namely a reduction in the standard weekly wage, seemed seldom to have the desired effect. Lord Higgins remarked that overworking was becoming, in some backward areas, a serious threat to society, more so, even, than crimes of theft and violence. He suggested that the more severe penalties usually reserved for serious crimes should be imposed on those convicted of overworking. In his experience, he said, six to twelve months of forced idleness was a sufficient deterrent for all but the most hardened criminals.

Lord Sneath then said he was greatly disturbed by the large number of able scientists emigrating to Russia. Russia, he pointed out, had not signed the Euro-American agreement and, in Russia, people could work as hard as they liked and were actually paid higher wages for more work. He suggested that further representation should be made to Russia to stop this appalling exploitation of slave labour.

Lord Ragg told the House that representatives of the medical and nursing professions, which, because of the importance of their work, had the unique privilege of being allowed to work seven days a week, had informed him that their members would be willing to work only five days a week, provided that they received an appropriate rise in wages. The House decided that, because of the very grave shortage of staff in the Health Service, they could not accept this very generous offer.

Lord Neath, Shop Steward for unmarried mothers, said that he was pleased to report that, since sex had been officially designated recreation rather than work, there had been a marked reduction in the illegitimate birth rate. On this happy note the House rose and members left for their well-earned weekend rest.

**JOHN CLUTTON-BROCK**

(Reprinted from "Anaesthesia Points West" by permission of the Editor, and of the Author).

